# Microbiome overview in swine lungs

**DOI:** 10.1371/journal.pone.0181503

**Published:** 2017-07-18

**Authors:** Franciele Maboni Siqueira, Esteban Pérez-Wohlfeil, Fabíola Marques Carvalho, Oswaldo Trelles, Irene Silveira Schrank, Ana Tereza Ribeiro Vasconcelos, Arnaldo Zaha

**Affiliations:** 1 Centro de Biotecnologia, Universidade Federal de Rio Grande do Sul, Porto Alegre, Rio Grande do Sul, Brazil; 2 Department of Computer Architecture, University of Malaga, Malaga, Spain; 3 Laboratório Nacional de Computação Científica, Laboratório de Bioinformática, Petrópolis, Rio de Janeiro, Brazil; The Ohio State University, UNITED STATES

## Abstract

*Mycoplasma hyopneumoniae* is the etiologic agent of swine enzootic pneumonia. However other mycoplasma species and secondary bacteria are found as inhabitants of the swine respiratory tract, which can be also related to disease. In the present study we have performed a total DNA metagenomic analysis from the lungs of pigs kept in a field condition, with suggestive signals of enzootic pneumonia and without any infection signals to evaluate the bacteria variability of the lungs microbiota. Libraries from metagenomic DNA were prepared and sequenced using total DNA shotgun metagenomic pyrosequencing. The metagenomic distribution showed a great abundance of bacteria. The most common microbial families identified from pneumonic swine’s lungs were *Mycoplasmataceae*, *Flavobacteriaceae* and *Pasteurellaceae*, whereas in the carrier swine’s lungs the most common families were *Mycoplasmataceae*, *Bradyrhizobiaceae* and *Flavobacteriaceae*. Analysis of community composition in both samples confirmed the high prevalence of *M*. *hyopneumoniae*. Moreover, the carrier lungs had more diverse family population, which should be related to the lungs normal flora. In summary, we provide a wide view of the bacterial population from lungs with signals of enzootic pneumonia and lungs without signals of enzootic pneumonia in a field situation. These bacteria patterns provide information that may be important for the establishment of disease control measures and to give insights for further studies.

## Introduction

Respiratory disease in pigs is associated with significant production losses and is considered one of the main obstacles for the swine industry. Clinical respiratory disease in pigs is often polymicrobial and multifactorial [1]. *Mycoplasma hyopneumoniae* is the etiologic agent of swine enzootic pneumonia and a major contributor to porcine respiratory disease complex [1]. This bacterium attaches to the cilia and surface of the epithelium with participation of adhesins, resulting in clumping, damage and loss of cilia [2]. The loss of cilia is thought to be important in the increased incidence of secondary bacterial infections. *M*. *hyopneumoniae* is distributed world-wide in the swine herds, and the infection by *M*. *hyopneumoniae* is commonly associated with immunostimulation and immunosuppression [1], with a complex interaction network delineating the persistence of this bacterium in the field.

Other mycoplasmas found in porcine respiratory system include the *Mycoplasma hyorhinis* and *Mycoplasma flocculare*. *M*. *hyorhinis* is related to polyserositis and arthritis [3], whereas *M*. *flocculare*, which is closely related to *M*. *hyopneumoniae* based on a 16S rRNA sequence and whole genome comparison [4], can adhere to cilia but no damage and disease has been associated with this species [2, 3]. Furthermore, there is evidence that the secondary bacteria that habit the respiratory tract, such as *Pasteurella* spp., *Haemophilus* spp., *Streptococcus* spp., can increase their virulence abilities following *M*. *hyopneumoniae* infection.

Metagenomic analysis in both human [5] and murine [6] respiratory tracts revealed a surprising bacterial variability, showing an immediate necessity of the understanding of this complex dynamics of microbial community in the animals. Considering the importance of the swine enzootic pneumonia and the limited knowledge about the bacterial species that inhabit the swine respiratory tract, we have performed a total DNA metagenomic analysis from lungs of pigs in a field condition, with suggestive signals of enzootic pneumonia and without any infection signals.

## Materials and methods

Samples were collected in a swine slaughterhouse with official sanitary inspection from Southern Brazil (29°53'12"S, 50°16'11"W). The pigs were from finishing operation herds, kept under intensive management conditions. The herds have a history of chronic but undefined respiratory problems. However, the animals selected for collection did not present any clinical signs of pneumonia. All the assessed lungs were scored by the inspection system, according to the extent of lesions typical for swine enzootic pneumonia infection expressed in terms of consolidated tissue: none, mild (<10% of the surface), moderate (10–30%), severe (>30%) [7]. Lungs from 20 pigs presenting suggestive macroscopic signs of enzootic pneumonia with mild (nine animals) to severe score (11 animals) were selected. Another 20 lungs without both swine enzootic pneumonia and infection macroscopic signs (none score) were also selected.

Sterile saline solution (20 to 30 ml) was introduced into the tracheal and lungs as a lavage. These materials were transported and immediately processed in the laboratory.

**Fig 1:**
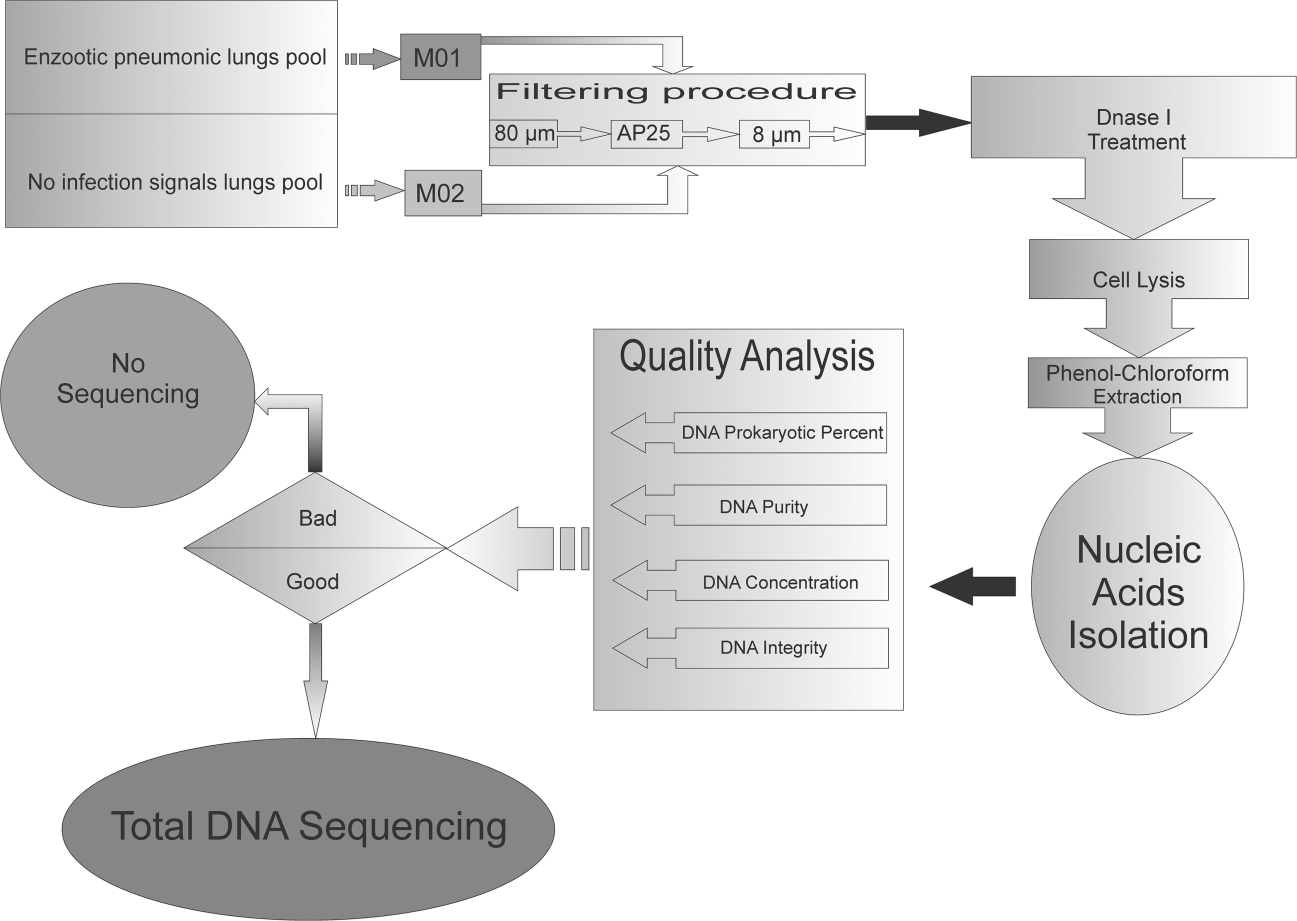
Workflow of samples preparation for metagenomic sequencing. The figure shows the samples collection; bacteria cell selection; bacteria DNA extraction and shotgun metagenomic sequencing. M01: tracheal and lungs lavage pool from 20 pneumonic lungs. M02: tracheal and lungs lavage pool from 20 lungs without both swine enzootic pneumonia and infection macroscopic signs.

The material from lungs with suggestive enzootic pneumonia signs and from lungs without macroscopic signs were pooled and named M01 and M02, respectively. Fig 1 shows the workflow of the sample preparation and metagenomic DNA extraction. Collected samples were treated at 37°C for 20 min with an equal volume of Sputasol (Oxoid, Hampshire, United Kingdom) to dissociate the mucus and where then passed through an 80-μm, AP25 and 8-μm filters (in this order) to remove large cells. The remaining material was then centrifuged and treated with 15,000 U/ml of DNase I at 37°C for 2 hrs to remove host DNA. After centrifugation, the pellets were resuspended in 10 ml SET buffer (20% sucrose, EDTA 50 mM, Tris-HCl 50 mM, pH 7.6), centrifuged, and the final pellet resuspended in SET buffer (2.5 ml). DNA was extracted from this cell suspension with phenol-chloroform protocol. Briefly, the resuspended cells were lyzed with 40 mg lysozyme, 1mg Proteinase K, SDS 10% followed by phenol-chloroform extraction. DNA concentration, purity, and the overall integrity were checked on the Quibt^TM^ (Invitrogen), NanoDrop (Thermo Scientific), and by agarose gel electrophoresis, respectively. The representation of prokaryotic and eukaryotic DNA was analyzed by semi-quantitative PCR assay using the 18S rDNA and 16S rDNA universal primers, with serial dilutions of the metagenomic DNA as template.

The DNA bacterial composition in both DNA samples was predicted by a parallel sample collection in a different swine slaughterhouse from another geographic location. Sample collection, processing and DNA extraction protocols were the same as described below. Furthermore, previously to metagenomic sequencing, we empirically tested the bacterial composition in both DNA samples by two rounds of a semi-quantitative PCR assay with serial dilutions of the extracted total DNA. The first round was performed with a specific set of primers, which we have designed, from 23S-rDNA region absent in mycoplasmas genomes (forward: 5’-GAACTGAAACATCTAAGTA-3’; reverse: 5’-GGTTTCAGGTTCTATTTC-3’). The other round-assay was tested with specific primer pairs to the gene MHP7448_0235, which encoded for myo-inositol 2-dehydrogenase, that is an exclusive gene of *M*. *hyopneumoniae* among the *Mycoplasmas* (5’-CGCCAAAAGCCTTAGAAGC-3’ and 5’-CAATAAACCCTTGTGCCCG-3’). These essays were performed to provide insights about the *M*. *hyopneumoniae* and other bacterial presences in the DNA samples, prior to total sequencing.

DNA shotgun metagenomic libraries of each pool (M01 and M02) were prepared by following the instructions from the Rapid Library Preparation Method Manual—GS FLX Titanium Series (454-Roche). Sample collection, metagenomic DNA preparation and pyrosequencing were performed in duplicates. Initially, sequence analysis and assembly were based in Newbler Assembler v.2.5.3. Inherent artifacts, replicates or low quality sequences were further filtered and removed with Replicates software [8] and LUCY program [9].

Two approaches were used to analyze the taxonomic distribution of each read hit. First, MG-RAST server [10] using the Lowest Common Ancestor (LCA) method and MEGAN4 [11], both with default parameters, were applied. Meta-RNA software was employed to perform 16S rRNA identification [12]. Secondly, the Metagenomics (MG) workflow [13] was applied, which performed different mappings restraining subsets of reads by distinct thresholds using 32 most prevalent genera reported by MEGAN4 with default parameters. The bacteria candidates were selected based on identity and coverage thresholds and expected values. The MG workflow threshold was a minimum of 50% identity and 50% coverage in the matches and a maximum expected p-value of 0.01. The MG workflow introduces a three-level mapping which allows users to not only discover blocking organisms but to also obtain quality distance measurements between the best ^n^ (usually^n = 3^) candidates for every match.

In order to validate the results of the experiment, a hypothesis contrast was carried out on the difference of the abundance of reads mapped to each species. In this sense, the probabilities of observing the reads abundance per species was computed and compared against the probability of such levels of abundance happening by chance. Probabilities were given in the form of p-values computed from the Z-score statistic of the ratio of reads-abundance per species. The resulting p-values were used to assess the confidence level of the outcome of the experiment, along with enabling a partial verification of the pooling method.

## Results

**Fig 2:**
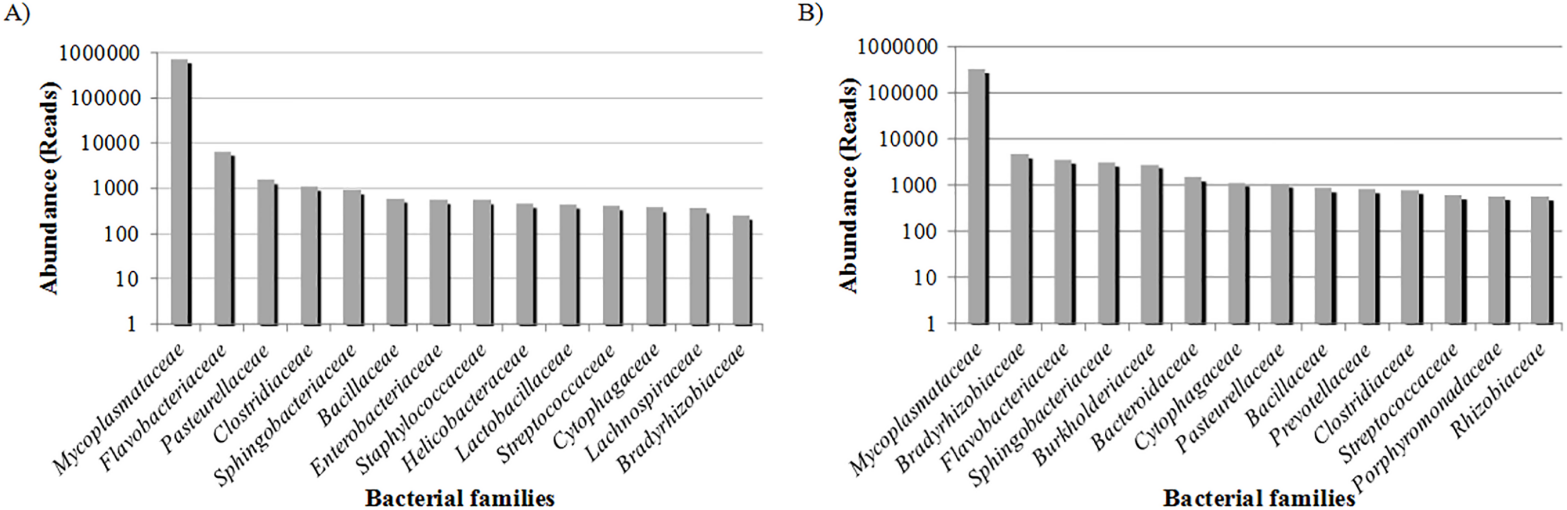
Most abundant bacteria families identified in the microbiome. (A) Most common families from enzootic pneumonic lungs (M01) group. (B) Most common families from carrier lungs (M02) group. The x-axes shows the most prevalent families matched in the samples. The y-axes show the abundance of reads from each of the identified families.

Total DNA from M01 (libraries from lungs with macroscopic signals suggestive of enzootic pneumonia) and M02 (libraries from lungs without signals of infection) yielded 79,998,248 and 48,482,448 base pairs (bp), respectively, and 80% of them were assigned to bacterial species. Bacteria domains represented 99% of the total sequenced reads, and the remaining reads were from the host (S1 Table). The average read length after trimming and filtering was of 482 bp and 453 bp for M01 and M02, respectively. Reads mapping was performed and allowed mapping of 678,500 reads from M01, and 581,383 reads from M02 metagenomes (S1 Table). The MG workflow mapping coverage is presented in S2 Table. The metagenomic distribution performed with MG-RAST and MG workflow showed a great bacterial abundance. Fig 2 shows the fourteen most prevalent families matched. The most common families identified from M01 sequences (MG-RAST ID mgm4530045.3) were *Mycoplasmataceae*, *Flavobacteriaceae* and *Pasteurellaceae* (Fig 2A), while in the M02 reads assignment (MG-RAST ID mgm4530046.3), the most common identified families were *Mycoplasmataceae*, *Bradyrhizobiaceae* and *Flavobacteriaceae* (Fig 2B).

**Fig 3:**
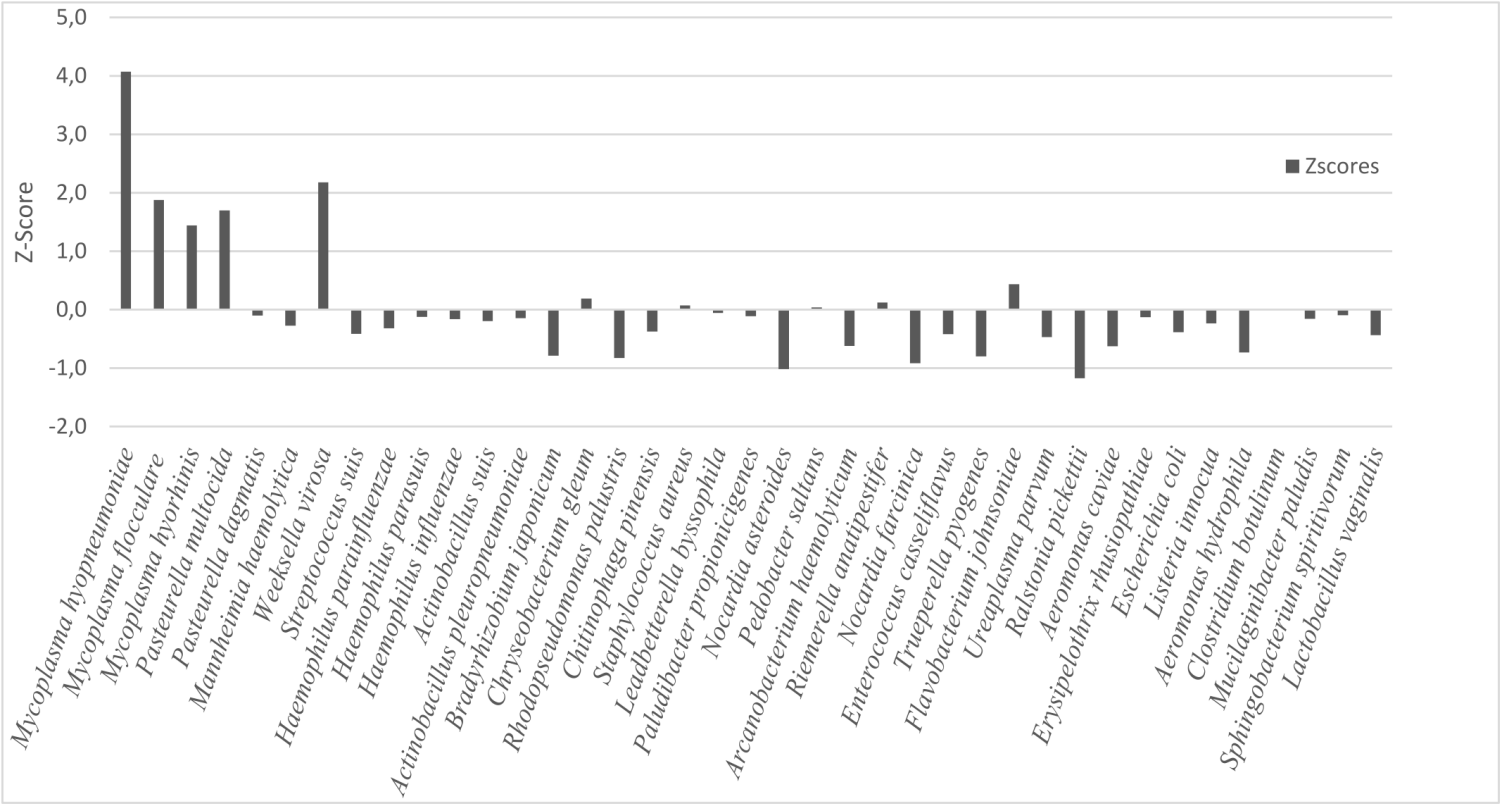
Z-score of the ratio of mapped reads between samples. The x-axis shows the most prevalent bacteria species reported. The y-axis shows the Z-scores from the normalized ratio of mapped reads.

In addition to the MG workflow results, two statistical analyses were further performed on the abundance of reads per species. Firstly, the Z-score test was used to enable the detection of variation across the samples. In order to calculate the Z-scores, read abundances per genome were normalized and converted to the same scale using a ratio of read abundances in the enzootic pneumonic lungs (M01) to carrier lungs (M02). The base 2 logarithm was used to balance the importance of small and large ratios (high and less represented species). The Z-score was computed by subtracting the mean and dividing by the standard deviation (Fig 3).

Secondly, we have computed the p-values of the Z-score test, to account for the statistical significance of the observed variations. To do this we start from the Z-score test. Since negative and positive Z-scores are equally important observations, the absolute value of the negative Z-scores is computed. This enables us to compute the right-tailed cumulative distribution function of a normal distribution N (0.1) with equal results for the same statistic ± z. At last, the resulting area is subtracted from 1 to obtain the final p-value.

**Fig 4:**
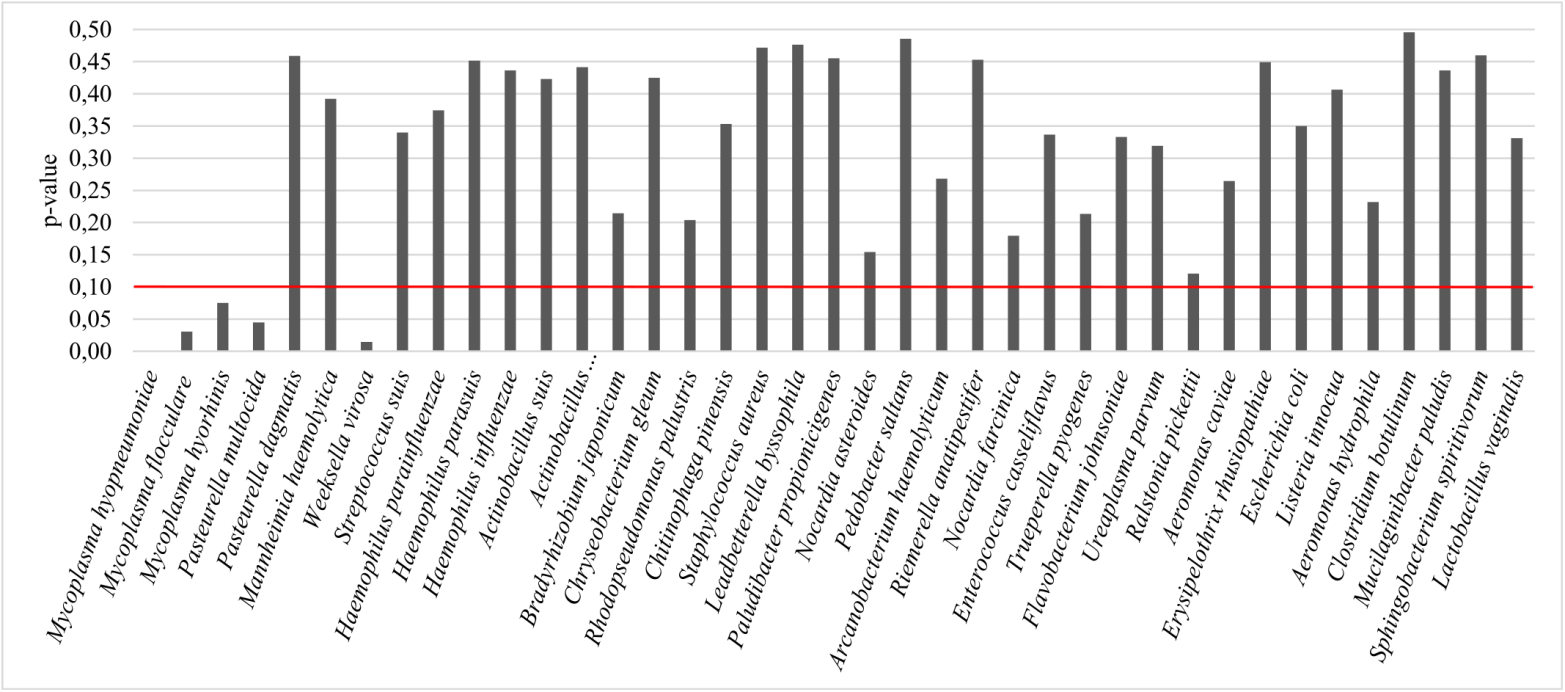
p-values of the ratio of mapped reads between samples. The x-axis shows the most prevalent bacteria species reported. The y-axis shows the p-values from normalized ratio of mapped reads. The red line represents the significant threshold set at an α = 0.10.

In Fig 4, the p-values of the ratio of mapped reads between the samples shows five species (*M*. *hyopneumoniae*, *M*. *flocculare*, *M*. *hyorhinis*, *Pasteurella multocida* and *Weeksella virosa*) whose statistical significance has remained relatively intact after pooling for an α = 0.10.

**Fig 5:**
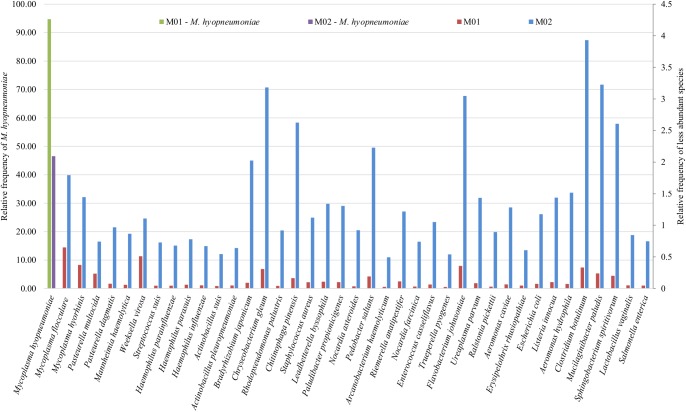
Mapped reads to the most abundant species identified. Normalized data points are separated into two y-axes, mainly one for the *M*. *hyopneumoniae* species and the other one for the rest of the species. In the x-axis, the most abundant species are shown. The y-axis shows the relative frequency in two scales; left: in green (M01) and purple (M02) the relative frequency scales for *M*. *hyopneumoniae*; right in red (M01) and blue (M02), the relative frequency scale for the remaining species.

The number of assigned reads to taxa is summarized in Fig 5, which shows that the reads distribution in the carrier lungs (M02) is more uniformly distributed than in enzootic pneumonic lungs (M01). Moreover, carrier lungs had a greater diversity of bacterial family population (Fig 2 and S1 File). Further, we have found some sample-specific bacterial families for both pneumonic (*Gallionellacea* and *Thermotogaceae*) and carrier lungs (*Alcaligenaceae*, *Neisseriaceae* and *Rhizobiaceae*) groups. The variety of species in swine carrier’s metagenome should be related to the normal flora from lungs and also to the secondary microorganisms that are able to invade the tract following *M*. *hyopneumoniae* infection.

## Discussion

The bacterial abundance of samples from swine lungs with macroscopic signals suggestive of enzootic pneumonia (M01) and from swine lungs without signals of infection (M02) was accessed by the shotgun metagenome approach.

Because of the difficulties in obtaining large quantities of DNA from individual lungs, we chose to prepare the DNA samples by pooling materials from 20 different animals. However, since pooling samples may generate a bias due to the predominance of several bacterial groups, our analysis and discussion were carefully oriented to minimize this bias, and we have obtained a great number of bacterial genera, which can indicate acceptable results. On the other side, it is also noteworthy that the pooling was performed with a considerable number of samples (20), thus improving the resemblance of the samples to that of the original population. The pooling of samples from different groups (e.g. control and case groups) has been subject of keen debate several times [14]. The discussion focuses mainly on the level of the impact on the statistical significance of averaged samples compared to original samples. In general, pooling affects the p-value of observing extreme events since pooling mostly produces a smoothness-effect, but the shared abundance levels across the samples remain mostly intact.

The MG workflow developed by Pérez-Wohlfeil et al. [13] was used to analyze the composition of the samples in order to detect the microbial species present. In this line, the metagenome analysis showed a large fraction of bacterial species diversity. *Mycoplasma* was the most predicted genus while *M*. *hyopneumoniae* the most predominant species representing almost 95% of reads from M01 and 47% of reads from the M02 (Fig 2A, Fig 2B, Fig 5).

Initially, these findings raised concerns about the sample preparation and representation. Therefore, we performed a bacterial composition estimative in both DNA samples by using a semi-quantitative PCR assay with serial dilutions of the total DNA. Using this approach we have concluded that *M*. *hyopneumoniae* is the most common species in the metagenomic DNA from swine’s lungs. These findings are supported by several other recent field studies, which have shown the high prevalence of mycoplasmas in swine lungs [15–17]. Therefore, metagenomic DNA from swine’s lungs are complex metagenomic samples, and despite the inherent technical difficulties with the extraction procedure, the sample pooling strategy allowed us to obtain a DNA sample of good quality. As expected, mycoplasmas were the most common microorganisms detected in both samples (M01 and M02) (Fig 2A, Fig 2B) as these bacteria are ubiquitous in the swine herds around the world [3]. The maintenance of these bacterial populations in the environment is associated with suckling piglets’ infection by their mother and by the spread of infection after weaning [18, 19]. Some animals may develop the disease and others will be asymptomatic carriers. Intrinsic factors, such as environmental, host and pathogen virulence will define if the disease will occur or not.

A detailed analysis at species level of some bacteria, such as *Mycoplasma* spp. from MG-RAST match was not enough to differentiate species from the same genus and therefore, using this approach (MG-RAST) *Mycoplasma* species abundance was not identified. However, the MG workflow [13] performed a detailed analysis, with custom parameters, which allowed an accurate differentiation between reads assignment for different *Mycoplasma* species, in addition to other bacteria species, resulting in a more specific identification. The overall comparison from both metagenomes indicated that carrier lungs presented a better reads distribution among bacterial species than pneumonic lungs (Fig 5). Although some differences exist between the results produced by MG-RAST (S1 Table) and MG-Workflow (Fig 5), these are due to both programs using rather different approaches to compare the metagenomes. MG-RAST performs a clustering of proteins from which prototypes are compared using sBLAT [20]. On the other hand, the MG-Workflow compares the full genomic material contained in the samples against a custom database using BLASTn. Furthermore, it becomes of interest from the scientific point of view to draw the conclusions from different software approaches.

*M*. *flocculare* did not appear as a lung bacterium species in our first analysis (MG-RAST); although, numerous studies showed *M*. *flocculare* presence in swine lungs [3, 16, 21]. However, a detailed analysis using the MG workflow [13] revealed reads assignment to *M*. *flocculare* (see Fig 5). Several state-of-the-art metagenomics classification software use the Lowest Common Ancestor (LCA) algorithm to assign reads to elder ancestor taxons when there is not a significant difference between the expected values in the best-reported matches. Thus, these approaches do not report high quality alignments when these are shared across pairs of species. The n-level option mapping available in the MG Workflow enabled us to detect that *M*. *hyopneumoniae* and *M*. *flocculare* shared approximately 75% of the found high quality alignments (Fig 5). In addition, the multiple level approach enabled us to find that between 0.6% and 2% of the reads were matched with slightly better expected value to *M*. *flocculare*. *M*. *flocculare* is a non pathogenic species from swine lungs [3] with genome composition closer to *M*. *hyopneumoniae* genome [4]. Therefore, reads matched to *M*. *hyopneumoniae* represent 95% of the enzootic pneumonic lungs and 47% of the carrier lungs’ metagenomes, while *M*. *flocculare* represents 0.6% of the enzootic pneumonic lungs’ and 2% of the carrier lungs’ metagenomes.

Earlier *M*. *hyopneumoniae* infection by vertical transmission from sow to piglet during lactation is suggested as the main transmission factor in piglets [18]. The infection can either be followed by clearance of the infection with bacterial maintenance in subclinical animals or be enhanced with subsequent prevalence of lung lesions at slaughter [18, 19, 22]. However, the airborne transmission is an important mechanism of *M*. *hyopneumoniae* reinfection and may occur between geographic closer farms [23, 24]. Several management and environmental factors have been shown to contribute to swine enzootic pneumonia, such as low general herd health, poor disease prevention, high pen stocking density, mixing pigs of different origin, and differences in the route of infection [25, 26].

Following contraction of enzootic pneumonia disease, an adequate treatment can promote the clearance of lung lesions, but *M*. *hyopneumoniae* may remain in the respiratory tract and the pigs are then called ‘carriers’. Therefore, previous infections will not be detected during slaughter [22, 27] and under the field experimental conditions there are no *M*. *hyopneumoniae*-free pigs.

Comparing the identification of *M*. *hyorhinis* in the metagenome results, we observed a significantly high frequency from the carrier bacteriome (Fig 5). Some analyses have indicated the relationship of *M*. *hyorhinis* with enzootic pneumonia and respiratory disease [3, 16, 28, 29], although this bacterium is also a normal flora inhabitant of the upper respiratory tract of pigs [3]. Therefore, our results support the hypothesis that *M*. *hyorhinis* is mainly present to pigs’ upper respiratory tract, being present in both samples.

*P*. *multocida*, which was identified in both samples (Fig 5), is commensal in the upper respiratory tract of pigs, and can also act as a primary or secondary pathogen in swine pneumonia [15, 28]. The higher frequency of *P*. *multocida* from enzootic pneumonia samples observed in the present study was also found by other studies [15, 28]. The microbiological comparison between lungs with pneumonia signs and without signs showed that *P*. *multocida* is significantly more represented in the diseased pigs [28], suggesting that this bacterium is most likely a secondary invading pathogen and aggravating the enzootic pneumonia [15].

Interestingly, the ratio of *M*. *hyopneumoniae*, *M*. *hyorhinis* and *P*. *multocida* detection is largely variable among the previous studies. Therefore, according to Sørensen et al. [30], the divergence depends on the identification methods used, differences in health status of the animals, management factors of the farms and seasonal variation. Alternatively it may simply reflect the complex nature of pneumonia in swine.

Our results demonstrated that *W*. *virosa* has a high prevalence in the enzootic pneumonic lungs as well as in carrier lungs. This bacterium belongs to the *Flavobacteriaceae* family and seems to be a saprophyte of the mucous membranes from healthy human and animals. Vela et al. [31] have isolated *W*. *virosa* from swine lungs with clinical signs of pneumonia. Moreover, a causal relationship with pathogenicity has been reported with pneumonia, bacteremia, peritonitis, and urinary tract infections in humans [32]. We highlight that the presence of this bacteria in the swine respiratory tract should be aware, mainly by its putative pathogenicity potential.

Approximately 50% of the sequences assembled were represented by bacterial species that were found overlapping in both sample groups. This profile can reflect the higher variability of the pig’s lung microbiota. Until now, the origin of the pig’s lung microbiota has been unknown. However, it is most likely to be in a state of flux with the environment. Barfod et al. [6] hypothesized that mice lung microbiota would be obtained from local environment and littermates influenced by handling by human, feed and water. Nonetheless, the age and other factors may be reflected in the dynamic microbiota distribution. According to Kim and Isaacson [33] the bacterial communities profile in pig guts change as the animals grow, and the variations of gut bacterial populations of swine are caused by a variety of factors including genetics, probiotics and the use of antibiotics.

Microbiome characterization has been carried out in a large number of studies by sequencing of the 16S ribosomal RNA subunit gene. Despite the advantages, there are some limitations in amplicon sequencing, as it may fail to resolve a substantial fraction of the diversity in a community given various biases [34–36]. Moreover, it is limited to the taxa analysis. In contrast, the shotgun metagenomic sequencing approach avoids the 16S sequencing limitations. Instead of targeting a specific genomic locus for amplification, all DNA sequences are independently sequenced. This approach results in DNA sequences (reads) that can be mapped to any genomic locations that will be sampled from taxonomically informative organisms.

In the present study, metagenomic sequences were used to characterize bacterial diversity of the lungs microbiota from pigs’ lungs. Analysis of community composition in lungs and trachea from pneumonic swine’s and carrier swine’s confirmed the high prevalence of *M*. *hyopneumoniae*. The interactions among respiratory pathogens during swine infections are numerous and complex, so the modes of action of pathogens can be additive or synergistic. We provide a wide view of the bacterial population from lungs with signals of enzootic pneumonia and lungs without signals of enzootic pneumonia in a field situation. The current data are primarily descriptive and are limited for use in correlating compositional changes with corresponding functional aspects in the animal. Thus, further studies using different approaches such as metatranscriptomics and metabolomics will be needed to elucidate the bacteriome interactions and their roles in carrier and disease situations. However, the knowledge of the bacteria prevalence patterns in the swine lungs is important for the establishment of disease control measures and to give insights for the further studies.

### Data submission

Metagenomes accession numbers given by MG-RAST in the present study were mgm4530045.3 (SwineLungs_H8T5UUH01_NR_MH_SS.trim) and mgm4530046.3 (SwineLungs_H8T5UUH02_NR_MH_SS.trim).

## Supporting information

S1 File Alpha diversity analysis and rarefaction curve of samples M01 and M02. (DOCX)

S1 Table Metagenome from swine lungs with PES and carrier lungs. (XLSX)

S2 Table Mapping coverage per sequence in samples M01 and M02 using the MG-workflow. (XLSX)
