# Supplementary material for: Microbiome overview in swine lungs
Source: PLoS One. 2017 Jul 18;12(7):e0181503. doi: 10.1371/journal.pone.0181503 (PMC5515459; doi:10.1371/journal.pone.0181503)
Supplement: S1 File — (DOCX) [file pone.0181503.s001.docx]

S1 File

## Alpha diversity analysis of samples M01 and M02

The alpha diversity test is used to represent and compare the mean diversity of the representation of species between samples. The alpha diversity test is usually performed using several diversity indices. For that purpose, we have used the most common ones, the Shannon and Simpson diversity index.

|  | Shannon diversity index | Simpson diversity index |
| --- | --- | --- |
| M01 | 0.4930182 | 0.2021232 |
| M02 | 1.1659773 | 0.3712496 |

Table 1. Alpha diversity analysis depicting Shannon and Simpson indices.

Table 1 shows the Shannon and Simpson indices of alpha diversity for the samples M01 and M02. Notice that in both cases, M01 has a much lower diversity index due to a single organism (*Mycoplasma hyopneumoniae*) taking up to 95% of the sample.

The Shannon diversity index is calculated as the negative sum of the proportions of each species present (relative frequency of appearance) multiplied by its natural logarithm, i.e.:


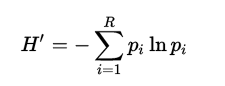


Then the Shannon entropy quantifies the uncertainty in predicting the species identity of an individual that is taken at random from the dataset (how “likely” it is that we are able to predict a random individual). In our case, M01 shows a smaller diversity value, since the amount of information (uncertainty in the composition of species) is much lower.

On the other hand, the Simpson diversity index is calculated as the somatory of the squared probability of finding each species at random, i.e.:


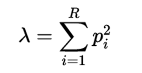


Thus $\lambda$ represents the chance of two individuals (taken at random) belonging to the same family.

In Figure 6 it can be seen that there exists a stronger diversity of species in M02, probably due to the fact that *Mycoplasma hyopneumoniae*’s presence is much lower compared to that of M01, where the species takes up to 95% of the DNA material.

## Rarefaction curve

We have calculated the rarefaction curve for samples M01 and M02 using a bootstrap method. Figure 1 shows the number of Taxonomical Units discovered (species) as a function of the size of the randomly drawn sample.


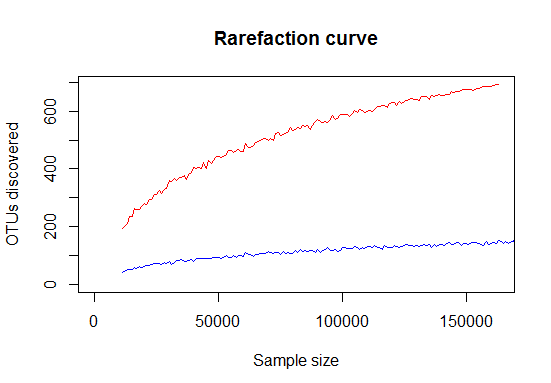


Figure 1. The rarefaction curve of the two samples. On the x-axis, the size of subsamples taken randomly from the sample population. On the y-axis, the number of OTUs discovered. The blue line corresponds to sample M01, whereas the red line corresponds to M02.

The rarefaction curve shows the discovering of species as a function of the number of subsamples taken from the population sample. A bootstrap approach was used to calculate it. In this particular case, M01 (blue line in Figure 1) shows a very fast asymptotical convergence, suggesting that the sample ideally represents the population, and that if the sample was bigger, almost no additional species would be discovered. On the other hand, in the case of M02 (red line in Figure 1) the opposite is observed: No convergence is achieved and thus the sample does not fully represent the population. More sampling would be needed in M02 to explore all existing species.
